# The location of unilateral axillary lymphadenopathy after COVID-19 vaccination compared with that of metastasis from breast cancer without vaccination

**DOI:** 10.1007/s11604-023-01387-1

**Published:** 2023-01-10

**Authors:** Kiyoko Mukai, Hiroko Tsunoda, Ryosuke Imai, Akiko Numata, Kumiko Kida, Ken Oba, Kazuyo Yagishita, Hideko Yamauchi, Naoki Kanomata, Yasuyuki Kurihara

**Affiliations:** 1 Department of Radiology, St. Luke’s International Hospital, 9-1 Akashi-Cho, Chuo-Ku, Tokyo, 104-8560 Japan; 2 Department of Pulmonary Medicine, St. Luke’s International Hospital, 9-1 Akashi-Cho, Chuo-Ku, Tokyo, 104-8560 Japan; 3 Department of Breast Surgical Oncology, St. Luke’s International Hospital, 9-1 Akashi-Cho, Chuo-Ku, Tokyo, 104-8560 Japan; 4 Department of Pathology, St. Luke’s International Hospital, 9-1 Akashi-Cho, Chuo-Ku, Tokyo, 104-8560 Japan

**Keywords:** COVID-19 vaccination, Unilateral axillary lymphadenopathy, Breast cancer, Metastatic lymphadenopathy

## Abstract

**Purpose:**

Unilateral axillary lymphadenopathy is known to occur after coronavirus disease (COVID-19) vaccination. Post-vaccination lymphadenopathy may mimic the metastatic lymph nodes in breast cancer, and it is challenging to distinguish between them. This study investigated whether the localization of axillary lymphadenopathy on magnetic resonance imaging (MRI) could be used to distinguish reactive lymphadenopathy after COVID-19 vaccines from metastatic nodes.

**Materials and methods:**

We retrospectively examined preoperative MRI images of 684 axillae in 342 patients who underwent breast cancer surgery from June to October 2021. Lymphadenopathy was defined as cortical thickening or short axis ≥ 5 mm. The axilla was divided into ventral and dorsal parts on the axial plane using a perpendicular line extending from the most anterior margin of the muscle group, including the deltoid, latissimus dorsi, or teres major muscles, relative to a line along the lateral chest wall. We recorded the presence or absence of axillary lymphadenopathy in each area and the number of visible lymph nodes.

**Results:**

Of 80 axillae, 41 and 39 were included in the vaccine and metastasis groups, respectively. The median time from the last vaccination to MRI was 19 days in the vaccine group. The number of visible axillary lymph nodes was significantly higher in the vaccine group (median, 15 nodes) than in the metastasis group (7 nodes) (*P* < 0.001). Dorsal lymphadenopathy was observed in 16 (39.0%) and two (5.1%) axillae in the vaccine and metastasis groups, respectively (*P* < 0.001). If the presence of both ventral and dorsal lymphadenopathy is considered indicative of vaccine-induced reaction, this finding has a sensitivity of 34.1%, specificity of 97.4%, and positive and negative predictive values of 93.3％ and 58.5%, respectively.

**Conclusion:**

The presence of deep axillary lymphadenopathy may be an important factor for distinguishing post-vaccination lymphadenopathy from metastasis. The number of axillary lymph nodes may also help.

## Introduction

Owing to the outbreak of coronavirus disease (COVID-19), COVID-19 vaccines have rapidly come into use worldwide. During a COVID-19 vaccine clinical trial, up to 16% of vaccine recipients complained of axillary pain and swelling as adverse reactions to the vaccine [1]. In fact, the number of vaccine recipients who were found to develop axillary lymphadenopathy, including subclinical axillary lymphadenopathy, was larger than that of recipients with axillary pain and swelling reported in the clinical trial. In a study by Park et al., breast ultrasonography (US) after COVID-19 vaccination revealed axillary lymphadenopathy on the side of the vaccination in as many as half of the recipients [2]. Retrospective radiology studies, which involved mammography, conventional computed tomography (CT), and fluorodeoxyglucose-positron emission tomography (FDG-PET), reported that the frequency of axillary lymphadenopathy after COVID-19 vaccination ranged from 3% to 66% [3–7].

Vaccination in the thigh or contralateral arm is encouraged in patients with a recent diagnosis of breast cancer [8]. However, this recommendation is generally not widespread for women, and vaccine recipients often receive the vaccine in the lateral arm on the side affected by breast cancer. Preoperative magnetic resonance imaging (MRI) in breast cancer often covers the axillary areas, as radiologists need to evaluate the presence of metastasis in the axillary lymph nodes. Recently, it has become challenging to distinguish breast cancer metastasis from unilateral axillary lymphadenopathy following COVID-19 vaccination.

Mori et al. reported a case of axillary lymphadenopathy that occurred after COVID-19 vaccination. In this case report, the largest lymph node was located deeper than the lower edge of the pectoralis major muscle on breast US [9]. In our experience, post-vaccination lymphadenopathy seems to occur deeper than breast cancer metastatic lymph nodes, which is consistent with the findings of this previous report. We hypothesized that lymphadenopathy after vaccination administered in the upper arm occurs deeper in the axilla due to the difference between the lymph flow from the breast and upper extremities.

Plaza et al. argued that morphologically abnormal lymph nodes are found at the higher levels (levels II/III), and normal lymph nodes are found at the lower level (level I) after COVID-19 vaccination [10]. However, to the best of our knowledge, since the publication of the aforementioned case reports, no detailed study was conducted to determine whether the trend is accurate in a large sample size. Therefore, the present study aimed to investigate whether the localization of axillary lymphadenopathy on MRI could be used to distinguish reactive lymphadenopathy after COVID-19 vaccines from metastatic nodes.

## Materials and methods

### Study population

The study examined 684 bilateral axillae on preoperative MRI images of 342 patients who underwent breast cancer surgery at our institute from June to October 2021. All participants included in this study were women and received a messenger ribonucleic acid (mRNA) COVID-19 vaccine (either a BNT162b2 vaccine [Pfizer–BioNTech, New York, NY, USA] or mRNA-1273 vaccine [Moderna, Cambridge, MA, USA]). Data on patient age and lymph node pathology, as well as vaccination details (date and injection site), were collected from electronic medical records. Lymph node metastasis was defined as the presence of isolated tumor cells (ITCs), micrometastasis, and macrometastasis detected via histological and cytopathological examinations of class V specimens obtained from ultrasound-guided needle biopsy conducted prior to chemotherapy. In morphological analyses, the shortest diameter of ≥ 5 mm is the most widely used size criterion for differentiating metastatic and non-metastatic lymph nodes on CT and MRI [11]. Lymphadenopathy was defined as cortical thickening or a short axis of ≥ 5 mm.

Patients with axillae with no lymph node metastasis of breast cancer and with lymphadenopathy on imaging in the axilla on the side administered with COVID-19 vaccine were assigned to the vaccine group. The metastasis group included patients with axillae with lymph node metastasis of breast cancer and with lymphadenopathy on imaging in the axilla on the side that was not administered with COVID-19 vaccine.

This single-institution retrospective study was approved by our institutional review board (21-R142).

### Imaging technique

MRI examinations were performed using 3.0-T (Discovery MR750w, GE Healthcare, Chicago, IL, USA), 3.0-T (MAGNETOM Verio, Siemens, Munich, Germany), and 1.5-T (Optima MR450w, GE Healthcare, Chicago, IL, USA) MRI systems with the patients in the prone position. All patients were examined with elevated upper extremities. The imaging protocol included one unenhanced and four dynamic post-contrast-enhanced axial T1-weighted images captured using three-dimensional gradient-echo sequences, as follows: (i) Discovery MR750w: 8-channel coil; repetition time, 5.2 ms; echo time, 2.1 ms; flip angle, 15°; slice thickness, 1.0 mm; slice gap, 0 mm; matrix, 384 × 330; and field of view, 350 mm; (ii) MAGNETOM Verio: 18-channel coil; repetition time, 4.0 ms; echo time, 1.5 ms; flip angle, 12°; slice thickness, 0.9 mm; slice gap, 0 mm; matrix, 384 × 384; and field of view, 350 mm; and (iii) Optima MR450w: 8-channel coil; repetition time, 8.0 ms; echo time, 3.3 ms; flip angle, 15°; slice thickness, 1.0 mm; slice gap, 0 mm; matrix, 360 × 350; and field of view, 360 mm. The contrast material (0.1 ml/kg gadobutrol [Bayer, Leverkusen, Germany] or gadoteridol [Eisai, Bunkyo-ku, Tokyo, Japan]) was injected intravenously, followed by a 20-ml saline flush at the rate of 1.5 ml/s. We evaluated axial, sagittal, and coronal images, including the images of four patients, which were obtained from other hospitals.

### Image analysis

**Fig. 1:**
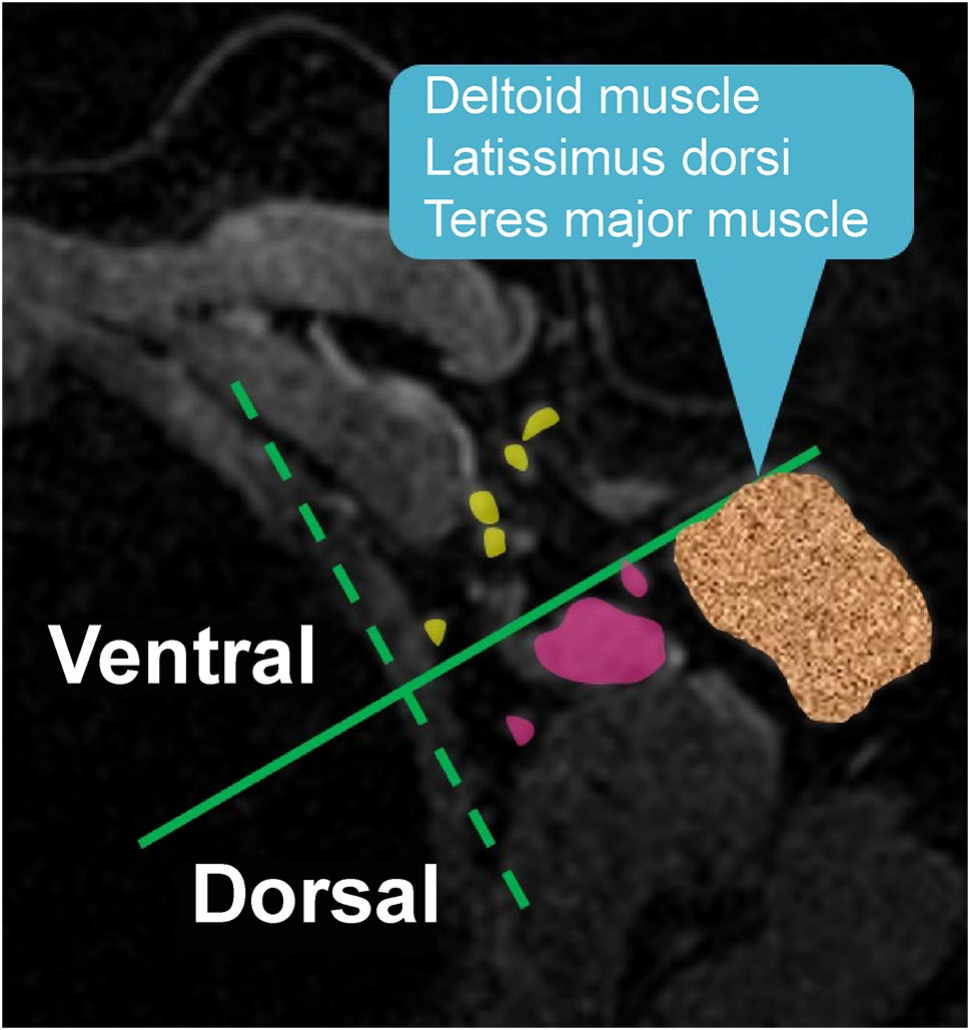
Division of axilla level I. Axilla level I was divided into ventral and dorsal parts on the axial plane using a perpendicular line extending from the most anterior margin of the muscle group, including the deltoid, latissimus dorsi, or teres major muscles, relative to a line drawn along the lateral chest wall

Axilla level I was divided into ventral and dorsal parts on the axial plane using a perpendicular line extending from the most anterior margin of the muscle group, including the deltoid, latissimus dorsi, or teres major muscles, relative to a line drawn along the lateral chest wall (Fig. 1).

The presence or absence of axillary lymphadenopathy, number of all visible lymph nodes, long and short diameters, presence of fatty hilum, and shape of lymphadenopathy (eccentric cortical thickening or round, oval, or horseshoe shaped) were evaluated. When axillary lymphadenopathy was present on both sides of the perpendicular line, the sites that occupied more than three-fourth of the area were recorded. When lymphadenopathy occupied one-fourth to three-fourth of both axillary areas, it was considered to be present in both areas, and the proportion of the region occupied in each area was recorded. The highest cortical thickness measurement or short axis of the axillary lymph node in each area was recorded in detail. Two radiologists with 3 and 30 years of experience in breast imaging retrospectively evaluated the MRI images. In case of discording opinions, a unified decision was reached after a discussion.

### Statistical analysis

All statistical analyses were performed using EZR (Saitama Medical Center, Jichi Medical University, Saitama, Japan), which is a graphical user interface for R (The R Foundation for Statistical Computing, Vienna, Austria) [12]. More precisely, it is a modified version of R commander designed to include statistical functions frequently used in biostatistics. The groups were compared using the Mann–Whitney *U* test for non-normally distributed continuous variables and Fisher’s exact test for two-category outcomes. *P* values were considered statistically significant at *P* = 0.05.

## Results

**Fig. 2:**
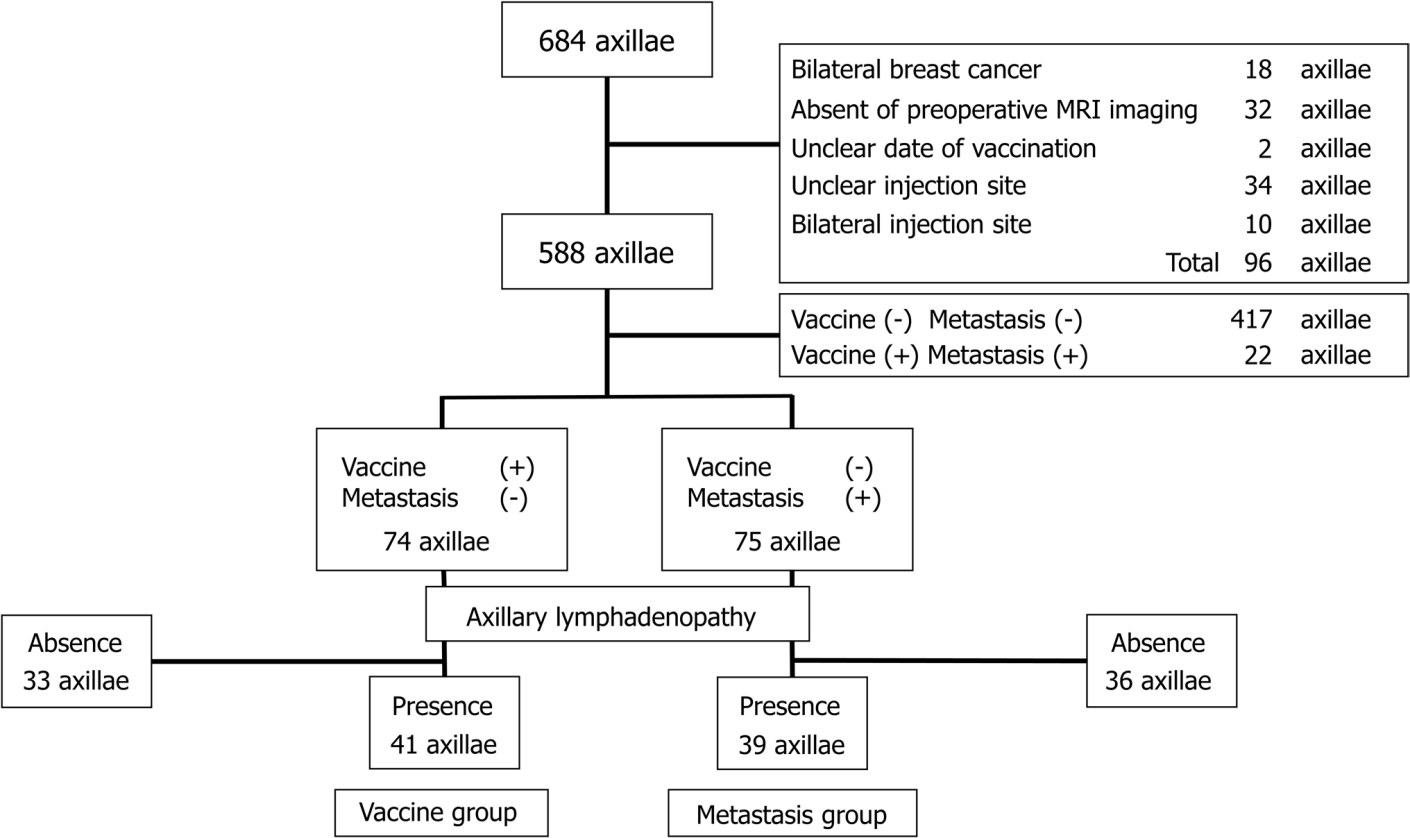
Participants in the metastasis and vaccine groups. Among 684 axillae (342 patients), 96 were excluded for the following reasons: simultaneous bilateral breast cancer, no preoperative MRI, uncertain vaccination date, uncertain upper extremity vaccination site, and vaccination in both upper extremities. Among the remaining 588 axillae, 22 axillae of patients who were vaccinated and had lymph node metastasis were excluded. We also excluded 417 axillae of patients who were not vaccinated and did not have lymph node metastasis. Of the 74 axillae of patients who were vaccinated and did not have lymph node metastasis, 33 with no lymphadenopathy were excluded. On the other hand, among the 75 axillae of patients who were not vaccinated and had lymph node metastasis, 36 axillae with no lymphadenopathy were excluded. Finally, 41 axillae were included in the vaccine group, and 39 were included in the metastasis group. *MRI* magnetic resonance imaging

Among the 684 axillae (342 patients), 96 were excluded because of simultaneous bilateral breast cancer etc. (Fig. 2). The vaccine group comprised 41 axillae (median patient age, 49 years; age range, 35–79 years), whereas the metastasis group included 39 axillae (median age, 47 years; age range, 28–82 years). MRI images were captured between the first and second vaccinations in the case of 18 out of 41 axillae in the vaccine group (no exact date was available for two of these axillae). The median time from the first vaccination to MRI was 15.5 (date range, 3–25) days. MRI images were captured after the second vaccination in 23 out of 41 axillae in the vaccine group (no exact date was available for one of these axillae). The median date from the second vaccination to MRI was 34.5 (date range, 6–126) days. Clinical characteristics of the metastasis group are presented in Table 1.

**Table 1:** Number of cases and definition of metastasis

	Metastasis group (*n* = 39)
Postoperative histopathological results	
ITCs	8 (2)^a^
Micrometastasis and macrometastasis	26 (7)^a^
Preoperative histopathological results	
Cytopathology (class V)	5^b^

ITCs, isolated tumor cells

^a^Patients who underwent surgery after chemotherapy

^b^Patients who underwent surgery after chemotherapy, and the postoperative histopathological results showed no lymph node metastasis

Lymphadenopathy-related findings according to the division presented in Fig. 1 were as follows.

The number of visible axillary lymph nodes in both areas was significantly higher in the vaccine group (median [range], 15 nodes [3–36]) than in the metastasis group (7 nodes [1–18]) (Table 2). The median and range of the short lymphadenopathy diameter in the ventral side of the axilla were significantly larger in the metastasis group (8.0 mm [5.5–22.5]) than in the vaccine group (6.4 mm [5.2–10.0]). The median long lymphadenopathy diameter in the ventral side of the axilla was also significantly larger in the metastasis group (14.1 mm [5.7–25.0]) than in the vaccine group (10.3 mm [5.6–24.9]). In contrast, there was no significant difference in the median short lymphadenopathy diameter in the dorsal axilla, as it was 6.6 mm (5.8–7.4) in the metastatic group and 6.0 mm (5.0–10.0) in the vaccine group. The long lymphadenopathy diameter in the dorsal axilla was also not significantly different: 9.0 mm (8.8–9.2) in the metastatic group and 10.3 mm (6.4–19.4) in the vaccine group. In terms of morphology, eccentric cortical thickening was more frequent in the vaccine group, whereas oval and round lymphadenopathy was seen more often in the metastasis group. There were no significant differences in the presence of fatty hilum.

**Table 2:** Characteristics of lymph nodes in the vaccine and metastasis groups

	Vaccine group (*n* = 41)	Metastasis group (*n* = 39)	*P* value
Lymphadenopathy			
Ventral			
Presence (%)	39 (95.1)	38 (97.4)	
Absence (%)	2 (4.9)	1 (2.6)	1^a^
Dorsal			
Presence (%)	16 (39.0)	2 (5.1)	
Absence (%)	25 (61.0)	37 (94.9)	< 0.001*, ^a^
Short diameter			
Ventral	6.4 mm (5.2–10.0)	8.0 mm (5.5–22.5)	0.004*, ^b^
Dorsal	6.0 mm (5.0–10.0)	6.6 mm (5.8–7.4)	0.673^b^
Long diameter			
Ventral	10.3 mm (5.6–24.9)	14.1 mm (5.7–25.0)	< 0.001*, ^b^
Dorsal	10.3 mm (6.4–19.4)	9.0 mm (8.8–9.2)	0.325^b^
Shape			
Ventral			
Eccentric cortical thickening (%)	27 (69.2)	16 (42.1)	0.068^a^
Round (%)	4 (10.3)	6 (15.8)	
Oval (%)	8 (20.5)	14 (36.8)	
Horseshoe shaped (%)	0 (0.0)	2 (5.3)	
Dorsal			
Eccentric cortical thickening (%)	13 (81.2)	1 (50.0)	0.405^a^
Round (%)	1 (6.2)	1 (50.0)	
Oval (%)	2 (12.5)	0 (0.0)	
Presence of fatty hilum			
Ventral (%)	21 (53.8)	20 (52.6)	1^a^
Dorsal (%)	8 (50.0)	1 (50.0)	1^a^
Number of visible lymph nodes			
Both areas	15 (3–36)	7 (1–18)	< 0.001*, ^b^
Ventral	10 (2–23)	6 (1–15)	< 0.001*, ^b^
Dorsal	3 (0–13)	1 (0–5)	< 0.001*, ^b^

Data are expressed as median (range)

*significant values

^a^Fisher’s exact test

^b^Mann–Whitney *U* test

**Fig. 3:**
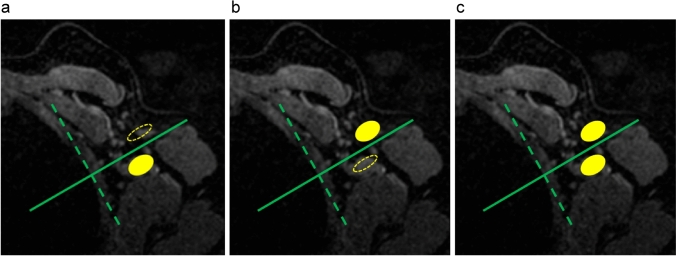
Patterns of lymphadenopathy according to area. Three patterns of lymphadenopathy were analyzed using the division shown in Fig. 1. **a** Lymphadenopathy in the dorsal area alone, **b** lymphadenopathy in the ventral area alone, and **c** lymphadenopathy in both areas

Dorsal lymphadenopathy was observed in 16 (39.0%) axillae in the vaccine group and in only two (5.1%) axillae in the metastasis group (Table 2). Ventral lymphadenopathy was observed in 39 (95.1%) axillae in the vaccine group and in 38 (97.4%) axillae in the metastasis group. As shown in Fig. 3, three patterns of lymphadenopathy were analyzed: (a) lymphadenopathy in the dorsal area alone, (b) lymphadenopathy in the ventral area alone, and (c) lymphadenopathy in both areas. Lymphadenopathy was seen in both areas in only one axilla in the metastasis group (1/39, 2.6%), while it was seen in both areas in 14 axillae (14/41, 34.1%) in the vaccine group; additionally, it tended to be located only on the ventral side in almost all axillae in the metastasis group (37/39, 94.9%) (Table 3). If the presence of both ventral and dorsal lymphadenopathy is considered indicative of vaccine-induced reaction, this finding has a sensitivity of 34.1%, specificity of 97.4%, positive and negative predictive values of 93.3% and 58.5%, respectively, and accuracy of 65.0% (Table 4).

**Table 3:** Presence of axillary lymphadenopathy

	Vaccine group (*n* = 41)	Metastasis group (*n* = 39)	*P* value
Pattern a (%)	2 (4.9)	1 (2.6)	1^a^
Pattern b (%)	25 (61.0)	37 (94.9)	< 0.001*, ^a^
Pattern c (%)	14 (34.1)	1 (2.6)	< 0.001*, ^a^

*significant values

^a^Fisher’s exact test

**Table 4:** Presence of axillary lymphadenopathy

	Vaccine group (*n* = 41)	Metastasis group (*n* = 39)
Showing pattern c (%)	14 (34.1)	1 (2.6)
Not showing pattern c (%)	27 (65.9)	38 (97.4)

**Fig. 4:**
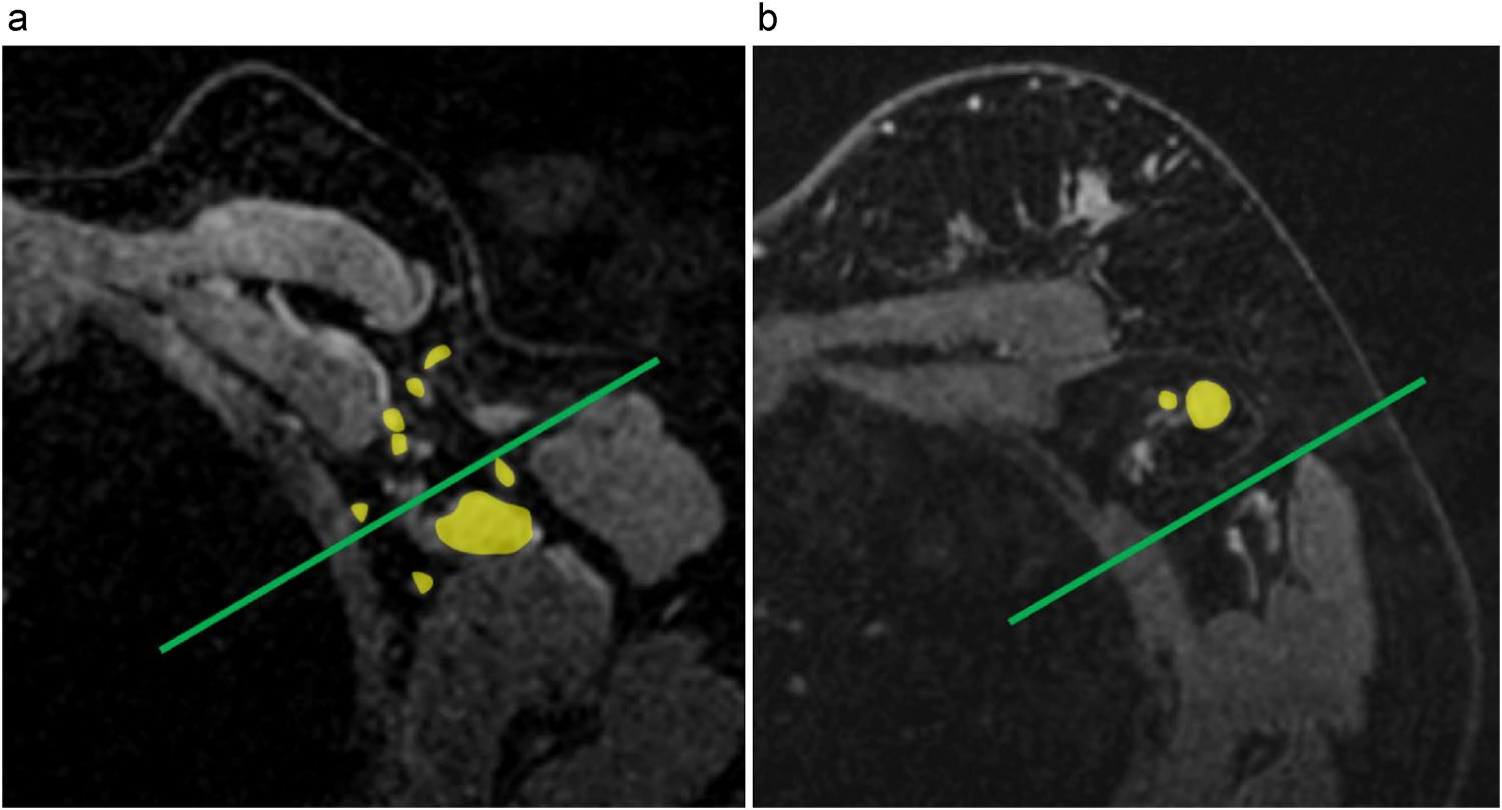
A representative MRI image of an axilla with post-vaccination lymphadenopathy and an axilla with lymph node metastasis. **a** Representative MRI image of the axilla with post-vaccination lymphadenopathy.** b** Representative MRI image of the axilla with lymph node metastasis. In the case of post-vaccination lymphadenopathy, significant lymphadenopathy was observed in the deep axilla. In the case of lymph node metastasis, lymphadenopathy was observed in the shallow axilla. *MRI* magnetic resonance imaging

Figure 4 shows a representative MRI image of an axilla with post-vaccination lymphadenopathy and an axilla with lymph node metastasis. In the case of post-vaccination lymphadenopathy, significant lymphadenopathy was observed in the deep axilla. The number of visible lymph nodes was also significant. In the case of lymph node metastasis, lymphadenopathy was seen in the shallow axilla. The number of visible lymph nodes was not as high as that in the axilla with post-vaccination lymphadenopathy.

## Discussion

Since the start of the widespread use of COVID-19 vaccines, there has been an increasing frequency of axillary lymphadenopathy seen on preoperative MRI in breast cancer, and it is challenging to distinguish metastasis from post-vaccination lymphadenopathy. Therefore, in this study, we aimed to distinguish between MRI images captured in two patient groups with the respective conditions and to identify essential elements that could aid in clinical practice.

When axilla level I was divided into the ventral and dorsal parts on the axial plane using a line drawn perpendicular to the chest wall, only ventral but not dorsal lymphadenopathy was found in most axillae in the metastasis group. Lymphadenopathy was found in the entire axillary region, including in the dorsal area, in a significantly larger proportion of axillae (34.1%) in the vaccine group than that in the metastasis group. These results suggest that MRI may be a valuable method for distinguishing metastasis from post-vaccination lymphadenopathy.

Using anatomical landmarks (the lateral thoracic vein as the vertical line and the second intercostobrachial nerve as the horizontal line), Clough et al. divided the lower part of the axilla (Berg’s level I and lower part of Berg’s level II) into four anatomical zones to localize the sentinel lymph node (SLN) [13]. In 98.2% (223/227) of the patients, the axillary SLN was located alongside the lateral thoracic vein. This area was equivalent to the ventral axillary area in this study. Breast lymphatic drainage is hypothesized to occur around the lateral thoracic vein, and metastatic lymphadenopathy tends to occur in the ventral axillary area.

Level I lymph nodes are divided into three groups: the pectoral (anterior), subscapular (posterior), and humeral (lateral) groups [10, 14, 15]. There are no previous reports on the lymphatic pathway from the deltoid muscle; therefore, details regarding this pathway are not yet known [16, 17]. Breast lymphatic drainage predominantly occurs into the pectoral (anterior) group of level I lymph nodes, while upper extremity lymphatic drainage predominantly occurs into the humeral (lateral) group of these lymph nodes. Lymphatic drainage from the breast and upper extremities initially follows a different pathway but then flows from the level II nodes to the level III nodes. This initial difference in lymph flow suggested that post-vaccination lymphadenopathy was located along the lymphatic drainage pathway from the upper extremities, which is deeper than that from the breast, and it was found to be predominantly located around the axillary arteriovenous system.

A review of axillary reverse mapping (ARM) serves to remind us that it is oncologically safe to preserve the ARM lymph nodes in patients with clinically node-negative breast cancer, who underwent a SLN biopsy, and in those with micrometastatic or macrometastatic lymph node involvement in the SLN, who were advised to undergo a complete axillary lymph node dissection, provided that there is no convergence between the ARM lymph nodes and SLN [18]. This review showed that some patients exhibited metastatic involvement of the ARM lymph nodes. Of note, the absence of lymph node enlargement in the dorsal axillary region in this study does not necessarily imply the absence of metastases. Theoretically, it may be possible to demonstrate that metastasis could be seen with a predominance of ventral lymph node involvement.

US is the primary modality used for the evaluation of axillary nodes, and breast MRI is useful for this evaluation as well [19]. US evaluates morphological criteria, such as cortical thickening and hilar effacement, which allows for the prediction of metastatic disease [20]. However, the morphological characteristics of post-vaccination lymphadenopathy include cortical thickening of > 3 mm, a round shape, the loss of fatty hilum, and cortical irregularity, which are overlapping and worrisome features [21–24]. These morphologic features are insufficient to distinguish metastatic lymphadenopathy from post-vaccination lymphadenopathy. MRI is a more objective and less operator-dependent modality than US [19].

This study has some limitations. First, this was a single-institution retrospective study. Second, in some cases, the axilla may not have been adequately included in routine preoperative MRI scans in breast cancer. Third, we were not able to confirm what happens to axillary lymph node enlargement postoperatively in cases in which the vaccination was administered contralateral to the location of breast cancer. Therefore, in such cases, although lymphadenopathy probably occurred due to the vaccination, this finding may not be completely grounded. Fourth, the time from vaccination to lymphadenopathy detection on MRI varied considerably. It has been reported that the median time from the first vaccination to lymphadenopathy detection on US was 9.5 days (range, 2–29 days) [25]. It was also reported that post-vaccination lymphadenopathy was found at 127 ± 43 days after the first vaccination [26]. In this study, the median period from vaccination to MRI was 14 days (range, 3–19 days) from the first vaccination and 38 days (range, 6–126 days) from the second vaccination; these time periods were considered appropriate. Despite the variations in the time periods, we were able to obtain the results presented in this study. Our results may be considered clinically useful for interpreting MRI images. Fifth, lymph node metastasis was defined as the presence of ITCs, micrometastasis, and macrometastasis; nonetheless, the effectiveness of ITC detection on images remains unknown. Although it is difficult to decide the exact factors that the definition of metastasis should be restricted to, we included patients with ITCs in the metastasis group because ITCs could also cause reactive lymphadenopathy. Sixth, all patients were examined with elevated upper extremities in the prone position. The EUSOBI guidelines recommend that both breasts be placed as deep as possible in the coil at the prone position [27]. The position of the upper arms is described as larger breast coverage obtained by placing both arms on the sides of the body without elevating the arms. Thus, some differences in the results depending on the position of the arms and the physical size of the patient are expected. However, the outcome of this study will be sufficiently helpful and applicable to any preoperative MRI for breast cancer taken with either elevated or lowered upper extremities. Seventh, we did not conduct a prospective clinical study to confirm our findings; therefore, further research is warranted.

## Conclusion

The presence of deep axillary lymphadenopathy and the number of axillary lymph nodes may be important for differentiating lymphadenopathy after COVID-19 vaccination and metastasis. This information could be valuable when deciding whether to use cytology or SLNs to confirm the presence of lymphadenopathy on preoperative MRI for breast cancer.
